# Tolerant macaque species are less impulsive and reactive

**DOI:** 10.1007/s10071-023-01789-8

**Published:** 2023-05-28

**Authors:** Louise Loyant, Bridget M. Waller, Jérôme Micheletta, Hélène Meunier, Sébastien Ballesta, Marine Joly

**Affiliations:** 1 Centre for Comparative and Evolutionary Psychology, Department of Psychology, University of Portsmouth, King Henry I Street, King Henry Building, Portsmouth, PO1 2DY UK; 2 Department of Psychology, Nottingham Trent University, Nottingham, UK; 3 UMR (7364), Laboratoire de Neurosciences Cognitives et Adaptatives, CNRS and Université de Strasbourg, Strasbourg, France

**Keywords:** Cognition, Evolution, Sociality, Executive function, Non-human primates

## Abstract

Inhibitory control, the inhibition of impulsive behaviours, is believed to be key in navigating a complex social environment. Species characterised by higher social tolerance, living in more complex groups, with more diverse relationships, face higher uncertainty regarding the outcome of social interactions and, therefore, would benefit from employing more inhibitory strategies. To date, little is known about the selective forces that favour the evolution of inhibitory control. In this study, we compared inhibitory control skills in three closely related macaque species which differ in their social tolerance style. We tested 66 macaques from two institutions (*Macaca mulatta*, low tolerance; *M. fascicularis*, medium tolerance; and *M. tonkeana*, high tolerance) using a battery of validated inhibitory control touchscreen tasks. Higher social tolerance was associated with enhanced inhibitory control performances. More tolerant species were less impulsive and less distracted by pictures of unknown conspecifics. Interestingly, we did not find evidence that social tolerance degree was associated with performance in reversal learning. Overall, our results support the hypothesis that evolution has promoted the development of socio-cognitive skills to cope with the demands related to the complexity of the social environment.

**Supplementary Information:**

The online version contains supplementary material available at 10.1007/s10071-023-01789-8.

## Introduction

Inhibitory control, the ability to override an impulsive, automatic or pre-learned response (Diamond 2013), is crucial in a complex social environment. A strong internal predisposition or an external distractor, tempting but counterproductive, irrelevant to the individual’s goal, must be overridden in order to do what is more appropriate or needed (Amici et al. 2008; Diamond 2013). Inhibitory control allows an animal to produce flexible responses and adjust behaviours that could interfere with social goals. For example, in a social context, an individual needs to constantly monitor its behaviours to maintain group survival and cohesion (Amici et al. 2008; Byrne and Bates 2007).

An understanding of variation in inhibitory control cross-species is crucial to identify the selective pressures acting on this ability. To date, we know little about the forces that affect the evolution of inhibitory control. Ecological factors (Amici et al. 2008; MacLean et al. 2014), brain size (Jelbert et al. 2016; Maclean et al. 2014) and social pressures (Amici et al. 2008; Johson-Ulrich and Holekamp 2020) have all been proposed to shape inhibitory control skills, but recent studies have challenged these claims (Jelbert et al. 2016; Kabadayi et al. 2017; Schubiger et al. 2020). Task choice and demands, physical understanding and experience have been shown to strongly influence inhibitory control performances in commonly used tasks (Kabadayi et al. 2017; Schubiger et al. 2020).

The organisation of the social environment is widely considered to be an important factor in the evolution of socio-cognitive skills (Amici et al. 2008; Byrne 1996; Dunbar and Shultz 2007; Shultz and Dunbar 2007; Wascher et al. 2018). On the one hand, the Machiavellian intelligence hypothesis suggests that in a despotic society, social manipulation and deception would lead to the development of richer, more developed socio-cognitive skills, such as inhibitory control (Byrne 1996). An individual living in a more competitive social environment would need to constantly inhibit inappropriate behaviours, such as feeding or mating, in the presence of higher ranked conspecifics (Amici et al. 2008; Byrne 1996). On the other hand, the social intelligence hypothesis (Dunbar and Shultz 2007; Shultz and Dunbar 2007) postulates that the demands associated with a complex, more cooperative social life generates selection for increased brain size and higher cognitive performances, including inhibitory control (Wascher et al. 2018). Both the Machiavellian and the social intelligence hypothesis have been supported in the literature, but preliminary data suggest that species living in more complex societies might have better socio-cognitive skills than other species (Joly et al. 2017).

Several definitions of social complexity have been proposed. For instance, Freeberg et al. (2012), suggested that complex social systems are those in which individuals frequently interact in many different contexts, with many different conspecifics, in many different ways. Bergman and Beehner (2015) defined social complexity as the number of differentiated relationships that individuals have with conspecifics. In complex societies, one individual may handle every conspecific differently based on their identity, their kinship, the social context or their life history (Bergman and Beehner 2015; Shultz and Dunbar 2017). In despotic species, individuals may treat their close kin differently from the rest of the group, but the rest of the group might be treated the same (Bergman and Beehner 2015; Shultz and Dunbar 2017). In such asymmetrical societies, the number of differentiated stable interactions is often lower than the size of the group (Bergman and Beehner 2015; Shultz and Dunbar 2017). In contrast, in tolerant species, the number of differentiated relationships often approaches the size of the group with a greater diversity of responses (Bergman and Beehner 2015). Interestingly, a recent study developed a social complexity index (closely related to the system uncertainty). This index was calculated using the following three factors: the social diversity in a group (the number of individuals, their age and sex), the flexibility in behavioural interactions (according to social situations) and the patterns of interactions between individuals (Rebout et al. 2021). Using the example of macaque societies, the researchers demonstrated that the complexity of a social system increased with social tolerance. Specifically, they argued that the less tolerant rhesus macaque’ societies were less complex than the highly tolerant Tonkean macaques’ societies.

Therefore, according to the social intelligence hypothesis, an individual living in a more tolerant society (defined as more complex) would need to employ more inhibitory strategies to engage successfully in more diverse social events occurring around them such as cooperation or coalition (Bergman and Beehner 2015; Diamond 2013; Fischer et al. 2017). Hence inhibition of impulsive behaviours and emotions would be crucial for living in a socially complex society (Amici et al. 2008; Diamond 2013; Wascher et al. 2018).

In a previous study, both high and low tolerance macaques were tested in a battery of tasks examining the physical and social domain of cognition (Joly et al. 2017). Results, however, were not conclusive. While macaques’ performances were similar within the physical domain, there was a difference in the social domain whereby the high tolerance species seem to perform better in tasks relevant for cooperation than the low tolerance species. The more tolerant macaque species also seemed to perform better in one basic inhibitory control task (the middle cup task, Joly et al. 2017). In the current study, we conduct a thorough test of the social intelligence hypothesis using a validated robust battery of tasks (Loyant et al. 2022), targeted to measure inhibitory control skills.

The macaque radiation is an ideal taxon for such a comparative study. Up to 23 species constitute the genus *Macaca* and are characterised both by a profound unity and a great diversity (Thierry 2007). These old-world monkeys have the broadest geographical range among non-human primates; widespread throughout Asia with a single species inhabiting northern Africa (Zinner et al. 2014). Macaques are mainly frugivorous, semi-terrestrial primates and they inhabit a wide range of habitats, from tropical to swamp forests and from seashore to semi-arid area (Zinner et al. 2014). Macaques share the same basic pattern of social organisation as they form multi male multi female groups of up to 100 individuals (Thierry et al. 2004). While males emigrate when reaching maturity, females remain in their natal group forming kin-bonded matrilines who maintain preferential relationships and support each other in conflicts (Thierry 2007). Beyond these shared basic features, however, macaque species differ greatly both in their morphology and in their styles of affiliation, aggression, dominance, nepotism and maternal behaviour (Balasubramaniam et al. 2012). Therefore, Thierry and colleagues (2004, 2007), proposed a classification of social style along a four-grade scale based, among other criteria, on patterns of aggression and reconciliation; from despotic and nepotistic style of social relationships to a more tolerant style with more open relationships.

Hence, the aim of this study was to explore the influence of social tolerance degrees on inhibitory control skills using a recently developed and validated battery of tasks (Loyant et al. 2022). Our main hypothesis is that the more tolerant species, arguably living in more complex social environments, will outperform the less tolerant species in the main components of inhibitory control*: inhibition of a distraction* (i.e., control of an emotional response to an internal or external distractor, in order to focus on a goal), *inhibition of action* (i.e., inhibition of a prepotent, unwanted, reflexive motoric action) and *inhibition of a cognitive set* (i.e., inhibition of a pre-learned cognitive set to flexibly adjust behaviours). We tested 66 macaques from three different species with different social tolerance degrees: rhesus macaques (*Macaca mulatta,* grade 1: low tolerance), long-tailed macaques (*M. fascicularis* grade 2: medium tolerance) and Tonkean macaques (*M. tonkeana*; grade 4: high tolerance). We hypothesised that the more tolerant a species is, the better the performance in all three domains of inhibitory control.

## Materials and methods

### Subjects

We tested 66 adult macaques from two institutions: the Medical Research Council Centre for Macaques (MRC-CFM) in Porton Down United Kingdom (UK) and the Centre of Primatology of the University of Strasbourg (abbreviated as CdP), France (FR).

In the MRC-CFM, 21 rhesus macaques (12 males and 9 females, aged from 3 to 17 years old, see Table 1) were housed in 14 different social groups with an average of 12 individuals per group. All animals were living in mixed groups consisting of one dominant male and several females, infants and juveniles (except for two unisex groups). Each group had access to an indoor free-roaming room (3.35 m × 8.04 m × 2.8 m) and an adjacent caged area (1.5 m × 6.12 m × 2.8 m), with a minimum total space of 3.5 m^3^ per breeding animal in the largest groups. All rooms were temperature controlled (20 °C ± 5) with humidity at 55% ± 10. Each free-roaming area had a large bay window at one end facing outdoors and allowing a natural day-night cycle. At the other end of each room was an internal window fitted with movable mirrors which the monkey could control using a handle, allowing them to view the activities outside their area. Rooms were enriched with climbing structures (platforms, poles, fire hose and ladders) and enrichment devices (food puzzles, boxes, plastic barrels and balls and small plastic blocks attached to structures or walls). Subjects received a supply of fruit and vegetables, dried forage mix (cereal, peas, beans, lentils etc.), bread and boiled eggs, in the morning and afternoon, with enough food to last for a 24 h period. All subjects had access to water and food prior to and during the experiment. Eighteen individuals had already participated in a behavioural study involving looking at pictures (Howarth et al. 2021) and all of them were familiar with basic training and clicker procedures. However, none of them had experience with touchscreen experiments. Thirty subjects (14 males, 16 females; aged from 3 to 17 years old) started the touch screen training phases but only 21 (12 males and 9 females, aged from 3 to 17 years old) successfully completed the training and were able to take part in the experiment (see Table 1).

**Table 1:** Description of the sex, the age and the ranking of the subject rhesus, long-tailed and Tonkean macaques

Total number			Rhesus macaques	Long-tailed macaques	Tonkean macaques
			28	20	18
Sex		Male	16	8	12
		Female	12	12	6
Age		Average	10	13.8	11
		S.D	5.7	2.9	5.6
		Min	2	7	4
		Max	25	21	23
Ranking	High	Male	13	6	5
		Female	8	5	0
	Low	Male	2	2	7
		Female	5	7	6

Average, S.D. (standard deviation), minimum and maximum of the values are given

In the CdP, all macaques were raised in social groups and had access to an indoor and outdoor area. Seven rhesus macaques (4 females and 3 males, aged from 2 to 25 years old) were raised in groups from 3 to 5 individuals consisting of one dominant male and several females and younglings. They lived in cages, measuring 16.5 to 33 m^2^ for the indoor area and 14 to 29 m^2^ for the outdoor area. Cages were enriched with climbing devices. Five tested subjects were naive to previous behavioural studies and cognitive experiments and two subjects had experience with touch screen cognitive experiments in their youth (Fizet et al. 2017).

We also tested twenty long-tailed macaques from the CdP (see Table 1). They were raised in social groups from 8 mixed groups of 2 to 13 individuals with one dominant male and several females and younglings. One group was constituted of only males and one group of only females. They lived in cages, measuring 16.5 to 33 m^2^ for the indoor area and 16.5 to 23 m^2^ for the outdoor area. Cages were enriched with climbing devices. Tested individuals were naive to any previous clicker training procedures and behavioural studies and experiments.

Finally, eighteen Tonkean macaques (see Table 1) were tested from two groups. Each group had free access to a wooded outdoor area, connected to a heated indoor area. Four individuals tested were from a group of five males. In this group, subjects had free access to an approximately 1364 m^2^ wooded outdoor area, connected to a 20m^2^ heated indoor area. These individuals were familiar with basic training and clicker procedures and they already took part in behavioural studies and experiments. They all had access to touch screen modules when they were young but were never tested with pictures (Ballesta et al. 2021). The other fourteen individuals were from a group of 21 to 23 individuals. In this group, subjects had free access to an approximately 3700 m^2^ wooded outdoor area, connected to a 20m^2^ heated indoor area. All individuals have free access to touch screen modules (Ballesta et al. 2021; Nioche et al. 2021). They were exposed to pictures of familiar conspecifics, but they have never been tested with pictures of unknown or threatening conspecifics. At the CdP, all animals were provisioned with commercial monkey pellets seven days a week, in addition to a supply of fresh fruit and vegetables once a week. Water was available ad libitum.

To ensure low stress levels, only subjects voluntarily interacting with the experimental setup participated in the study and were free to leave the testing area at all times. They were never isolated from other members of their group. The subjects had to undergo a training procedure to learn to use the touchscreen (see Online Resource S1).

The rank of each individual was calculated using David’s Scores (David 1987; Neumann et al. 2011) and Elo-ratings scores (a ranking method developed by Elo 1978; see De Vries et al. 2006) obtained from observations of agonistics interactions (see Online Resource S2). To increase the power of our analysis, the subjects were categorised as either high or low ranking. We considered high ranking individuals the two subjects at the top of the hierarchy at the time of the testing.

### Battery of inhibitory control tasks

#### Apparatus and procedures

**Fig. 1:**
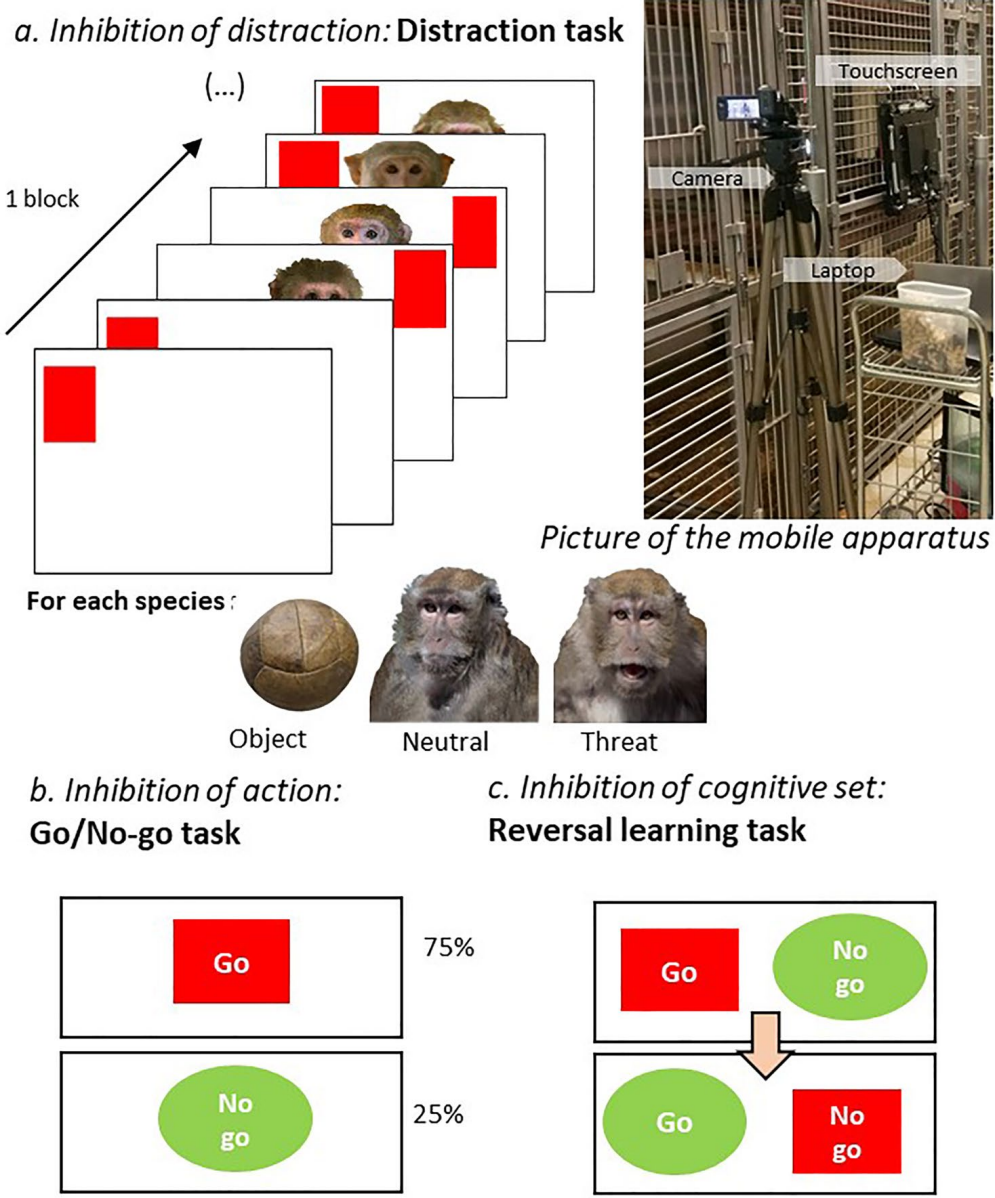
Picture of the apparatus (source: Louise Loyant) and schematic representation of the battery of inhibitory control tasks. **a** The Distraction task (*inhibition of a distraction*, a testing block of six trials is presented, a session is composed of 6 blocks with 3 different types of pictures as distractors), **b** the Go/No-go task (*inhibition of an action*), and **c** the Reversal learning task (*inhibition of a cognitive set*) are presented

For the experimental tasks, the set up was customised to be transported from one cage to another (see Fig. 1). Outside the cage, a laptop was connected to a capacitive touchscreen (ELO^®^ 1590L, frequency of 60 Hz, 19″ in diagonal, resolution 1280 × 1024 pixels). The program ELO touch solution^©^ 6.9.20 was used for calibration. The touchscreen was attached to the cage bars and the position was adjusted to each species and to each individual. All experimental procedures were carried out using MATLAB (version R2018b, using Psychtoolbox-3.0.15 functions), under Windows 10. The MATLAB custom scripts were specifically conceived for the needs of this study to record response latency and the success/failure of an answer; an individual progression file allowed the experimenter to abort and come back to the same point of a running session. If a trial was aborted the response latency was not recorded. The computer gave auditory feedback in response to the subject’s performance. All sessions were videotaped with one digital video camera (Sony HDR-CX330EB). In cases where more than one individual per cage was tested, or when other individuals from the group tried interacting with the touch screen, research assistants distracted the macaques who were not being tested at the time with food at the opposite side of the cage.

The subject initiated—or restarted—a session by touching a red cross. The session was paused if the subject exceeded the time limit (see each task for specific time limits). The session could also be aborted if the subject was not focusing its attention on the task (i.e., if the individual did not restart the paused session) or if other conspecifics interacted with the touchscreen. If the subject stayed inactive for more than 5 min the experiment was stopped and continued the next testing day, if the subject did not participate for three testing days in a row the subject was excluded from the task. The rewards (dry raisins) for each correct answer were given by hand.

#### Design

##### Inhibition of a distraction: Distraction task

With the Distraction task, we wanted to measure how macaques managed their response to distractors while doing a task. The subjects had to touch a red rectangular target presented at the same time as a central pictorial distractor (see Fig. 1a and Online Resource S3 for details). Subjects were presented with either “Control” trials (target presented alone), “Object” trials (target plus neutral object), “Neutral” trials (target plus a face of unknown conspecific with a neutral facial expression) and a “Threatening” trials (target plus a face of unknown conspecifics displaying an open mouth threat). A block was constituted of two “Control” trials followed by four trials with different pictures from the same category (one block was repeated twice). One session was constituted of 6 blocks with two blocks per category of stimulus. Each subject was tested in 3 sessions of 36 trials. Each block and trial were counterbalanced across subjects. We predicted that species with lower tolerance will have a stronger behavioural response to the distraction stimulus, particularly to the threatening stimuli and, therefore, have a longer response latency to the target stimulus compared to the other species of macaques.

##### Inhibition of an action: Go/No-go task

With the Go/No-go task, we wanted to investigate whether the more tolerant subjects will be better at controlling their impulsive action. Subjects were rewarded for touching a red rectangular “Go” stimulus (presented in 75% of the trials) and for withholding touching a green circular “No-go” stimulus (i.e., controlling an impulsive action). If the “No-go” stimulus was touched, the subjects received no reward and a time out. Each subject was tested in 5 sessions of 40 trials (see Fig. 1b and Online Resource S4 for more details). We hypothesised that the more tolerant species will be better at controlling their impulsive action and will less frequently touch the No-go stimulus.

##### Inhibition of a cognitive set: Reversal learning task

With the Reversal learning task, we assessed how macaques with different degrees of social tolerance control an automatic pre-learned response to learn a new rule. At the beginning of the task, two stimuli, a red rectangular “Go” rewarded stimulus and a green circular “No-go” unrewarded stimulus, were displayed simultaneously on the screen at counterbalanced locations (left or right of the screen, see Fig. 1c). When the subject touched the “Go” stimulus, the subject received a reward and a new trial began (acquisition rule). If the subject touched the incorrect stimulus the subject did not receive a reward and the two stimuli stayed on the screen until the correct stimulus was touched. If the background was touched nothing happened. A session consisted of 40 trials. Once a criterion of success was achieved (75% of correct trials out of 20 trials, i.e., the subjects touched the correct stimulus from the first attempt), the rule was reversed: the correct stimulus became the incorrect and the incorrect the correct. The reversed session was continued until the success criterion was reached again (75% of success for the whole session, see Online Resource S5 for more details). We hypothesised that the more tolerant species will learn the rules more quickly and will be less distracted by the previously learnt rule when learning the new rule.

As in previous batteries of tasks in animals (Beran and Hopkins 2018; Herrmann et al. 2010) the order of tasks was the same for all subjects. Although this design cannot eliminate the possibility of order effects (i.e., the participation on a given task affects performance on subsequent measures), it ensures consistency across subjects. Besides, we wanted our subject to have the same experience with inhibitory control testing as this ability can be learned and is directly influenced by previous inhibitory control testing (Diamond 2013; Schubiger et al. 2020).

### Statistical analysis

To study the relationship between the outcome variables of inhibitory control (see below for specific task outcomes) and the different degree of social tolerance (low, medium and high tolerance), we conducted LMM (linear mixed models, for repeated continuous outcomes) or GLMM (general linear mixed models, for binomial outcomes) using the R package ‘lme4’ v. 1.1–21 (Bates et al. 2015).

For the model analysis of the Distraction task, the outcome of the LMMs was the *Distraction control score*, representing the difference between the mean response latency to the target in “Control” trials for each individual (baseline) minus the mean response latency in the trials with pictures. A higher score indicates better control of a distraction, as the subject’s reaction to the stimuli interfered less with the goal of the task. A lower score indicates a higher distraction and thus a lower inhibitory control. We applied the following transformation to normalise the Distraction score (as advised in Field et al. 2012; Tabachnick, et al. 2007 for response latencies):

$$\text{Normalised Distraction control score}=\surd ((\text{max}(\text{Distraction control score}+1))-\text{Distraction control score})$$

For the Go/No-go task the outcome was the *success* in a trial when a “No-Go” was presented (i.e. not touching the No-go stimulus). A higher probability of success for No-go trial would indicate an individual was better at inhibiting the action. For the Reversal learning task, when taking both rules together, the outcome was the number of trials the subject needed to learn the rules.

As we previously demonstrated that the factors sex and age influenced inhibitory control performances (Loyant et al. 2021), we decided to include them, for each task, as controlling factors. As in the low tolerance species individuals were from two different institutions, we ran a preliminary analysis to test for this confounding factor. We did not find an effect of institution on the inhibitory control scores (see Online Resource S6), so we pooled all of the individuals from the low tolerance species together. The last explanatory variable category was experimental factors to control for habituation and learning: trial number, session number and the type of stimulus (the type of picture for the Distraction task and the reversed or acquisition rule for the Reversal learning task). The random factor of individual identity remained in all models to account for repeated measures of individuals. We used the functions ‘hist’ and ‘qqnorm’ (from the R package ‘stats’ v. 3.6.2) to visually check for the normal distribution of the residuals. For binomial distribution we used the function ‘simulateResiduals’ (from the package ‘DHARMa’). Models were compared by the likelihood ratio test using the function ‘anova’ from the R package ‘car’ v. 3.0–6. We applied backward reduction to analyse the contribution of each variable on the models (Field et al. 2012; Tabachnick et al. 2007). Initially, all explanatory variables and interaction were fitted in the maximal model. Non-significant interaction and terms on the model (*P* > 0.05) were dropped sequentially in *P* value decreasing order to simplify the model. Once an optimum model was obtained with only variables having a significant effect on the model, we compared the effect of each variable by comparing the optimum model and the model without this variable. We also used the post-hoc test Tukey’s Honest Significant Difference test (Tukey HSD test, function ‘glht’, package ‘multcomp’, version 1.4–18, accounting for multiple comparisons), to analyse specifically the difference between species with different degree of tolerance and between each type of stimulus. We ran a preliminary analysis to confirm that, before starting this first task, all species tested had similar response latency (see Online Resource S7).

In the Distraction task, to have a proxy of the behavioural response of the subjects, we also looked at the number of facial expressions displayed toward each type of trials (‘bared teeth’, ‘open mouth teeth’, ‘teeth chattering’, ‘lip smacking’). We used zero-inflated regression models (function ‘zeronfl’ from the package ‘pscl’, version 1.5.5,) via maximum likelihood to analyse the effect of social tolerance or the type of picture on the number of facial expressions displayed by the subjects. We compared the model obtained to a null model without the predictor using a chi-squared test on the difference of log likelihoods (function ‘pchisq’ from the package ‘stats’).

In the Go/no-go task we also used the Wilcoxon test (function ‘wilcox.test’, package ‘stats’, version 3.6.2) to compare species’ performances to the chance level (> 50% of probability of success) for the last session.

In the Reversal learning task, we also looked at the effect of social tolerance on each rule separately. For the acquisition rule, we applied a Kruskal–Wallis rank sum test as the variances were not homogenous (Levene’s Test *P* = 0.046). For the reversed rule we applied Anovas (as the variance was homogenous, Levene’s test *P* = 0.42).

## Results

### Results for the *inhibition of a distraction*: Distraction task

There was a significant main effect of the session (*χ*^2^_1_ = 70.33, *N* = 66, *P* < 0.0001) on the Distraction control score when all the sessions were taken together (see Online Resource S8). As the session number increased the subjects were getting habituated and less distracted by the stimuli. So we chose to analyse the Distraction control score for the first session to capture the macaque’s initial emotional reaction.

**Fig. 2:**
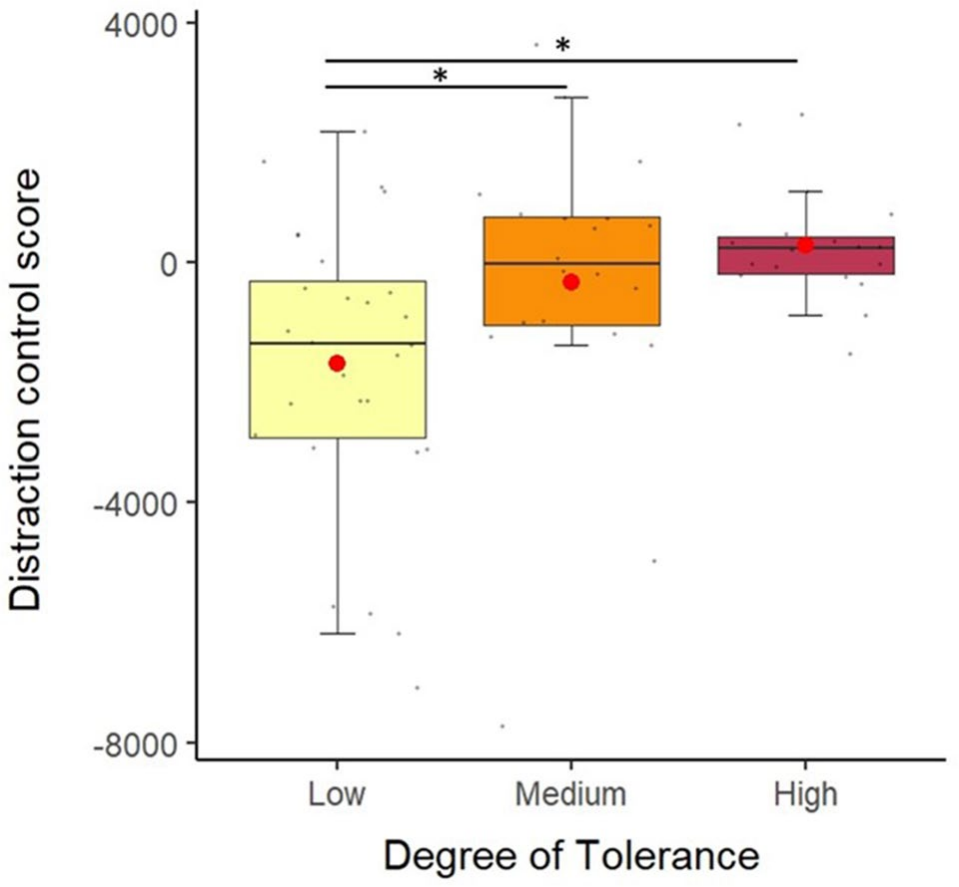
Mean Distraction control score (not normalised) between the low, medium and high tolerance species for session 1 of the Distraction task. Low tolerance species had a lower Distraction control score than the medium tolerance species and the high tolerance species. The red dot represents the mean per tolerance degree. Horizontal lines represent the 25th, 50th and 75th percentile and whiskers extend to 1.5 times the interquartile range. Black dots represent the mean for each individual *P < 0.05 (from the Tukey Post Hoc test) (color figure online)

For the first session, there was a significant main effect of the tolerance degree on the normalised Distraction control score (*χ*^2^_2_ = 9.857, *N* = 66, *P* < 0.01). The low tolerance species had the lower Distraction control score (raw Distraction scores here, *M* =  − 1279.12 ms, S.D. = 6396.71), compared to the medium tolerance species (*M* =  − 235.24 ms, S.D. = 6814.77) and the high tolerance species (*M* = 293.29 ms, S.D. = 4133.27, see Fig. 2 and Online Resource S9). From the Tukey Post Hoc test (see Online Resource S10), we found the same difference with the low tolerance species having lower score from the species with the intermediate level of tolerance (*Z* =  − 23.675, *P* < 0.05) and the high tolerance species (*Z* =  − 2.47, *P* < 0.05). Overall, a lower distraction score in the low tolerance species indicates a higher distractibility and thus a lower control of a distraction (see Table 2 for a summary of the results).

**Table 2:**
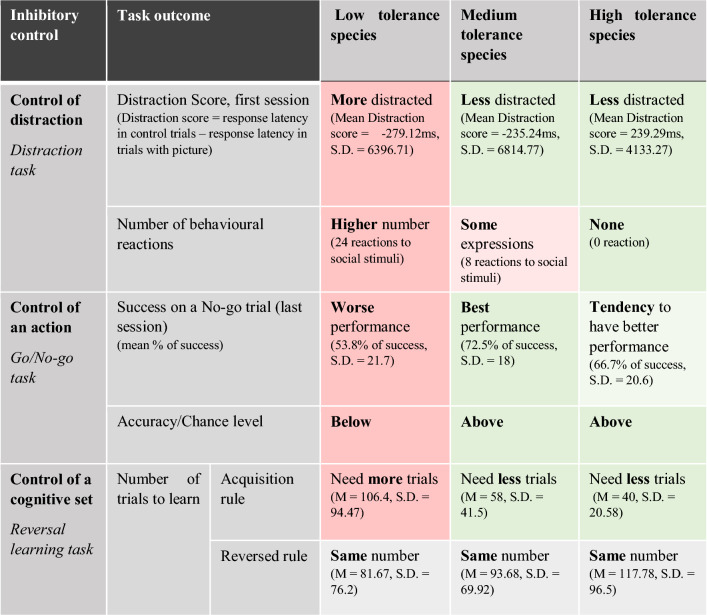
Summary of the results of the 3 tasks for low, medium and high tolerance species (average (M), standard deviation (S.D.))

The type of picture presented during a trial also had a significant main effect on the Distraction control score (*χ*^2^_2_ = 30.506, *N* = 66, *P* < 0.001). The Distraction control score for the trials with pictures (raw Distraction scores *M* =  − 69.51 ms, S.D. = 5602.427) were lower than Distraction control scores of “Control” trials (*M* = 151.24 ms, S.D. = 5206.49). Distraction control scores of trials with pictures of objects (*M* =  − 217.1 ms, S.D. = 5485.02) were higher than Distraction control scores of trials with pictures of a face (*M* =  − 827.8 ms, S.D. = 6363.585). Finally, Distraction control scores of trials with a threatening stimulus (*M* =  − 1653.67, S.D. = 7213.77) were lower than Distraction control scores of trials with neutral stimulus (*M* =  − 655.47 ms, S.D. = 6252.41). When looking at results of the Tukey HSD test (see Online Resource S11), the “Threatening” stimulus has the most distracting effect with a lower Distraction control score than “Control”, “Object” and “Neutral” trials. “Neutral” trials also had a lower Distraction control score than “Control” trials. Overall the threatening valence (the degree of positivity or negativity of a stimulus, Blackett et al. 2017) of the pictures had the most distracting effect. The other explanatory factors (age, sex and trial) did not have a significant effect on the Distraction control score.

#### Behavioural reactions

The number of behavioural reactions in response to stimuli (“bared teeth”, “lip smacking”, “teeth chattering”) depended on the type of picture. Twelve individuals from the low tolerance species reacted 18 times to the “Threatening” conspecific faces and 6 times to the “Neutral” conspecific. Four individuals from the medium tolerance species reacted 4 times to “Neutral” conspecific faces and 4 times to “Threatening” conspecific faces. The high tolerance species never reacted to pictures of conspecifics. None of the individuals reacted to “Control” trials with no pictures and “Object” trials (see Online Resource S12).

There was a main significant effect of the degree of social tolerance on the number of behavioural reactions (Chi-squared test, Log likelihood *P* < 0.01). When looking at each species separately we found, thanks to the Tukey post-hoc test, that the low tolerance species displayed more behavioural responses than the high tolerance species (see Table 2 and Online Resource S12). Medium tolerance species produced some behavioural reactions but there was no significant difference with the other species.

There was also a main significant effect of the type of picture on the number of behavioural reactions (Chi-squared test, Log likelihood *P* < 0.001). When looking at each type of picture separately (see Online Resource S12) we found that the individuals made more behavioural reactions toward the “Threatening” stimulus compared to “Object” trials or compared to “Control” trials.

Overall low tolerance species had the worst performances in all measurements of the Distraction task. They were more distracted and reacted more to a social distractor (particularly the threatening ones). These behavioural responses were mainly submissive responses (silent bared-teeth, avoidance, crouching, Thierry 2007; Thierry et al. 2004) which could reflect a higher emotionality (i.e. a measure of an individual’s emotional reactivity to a stimulus). The high tolerance species had the best performance; they controlled their behavioural response in all measurements with no visible reaction (even from the four individuals naive to pictures). The picture was intermediate for the medium tolerance species, they were not distracted by the distractors while performing the task, but they still demonstrated behavioural reactions toward social pictures which could be associated with a moderate emotionality. Two rhesus macaques were not willing to participate in further testing after this task.

### Results for the *inhibition of an action*: Go/No-go task

**Fig. 3:**
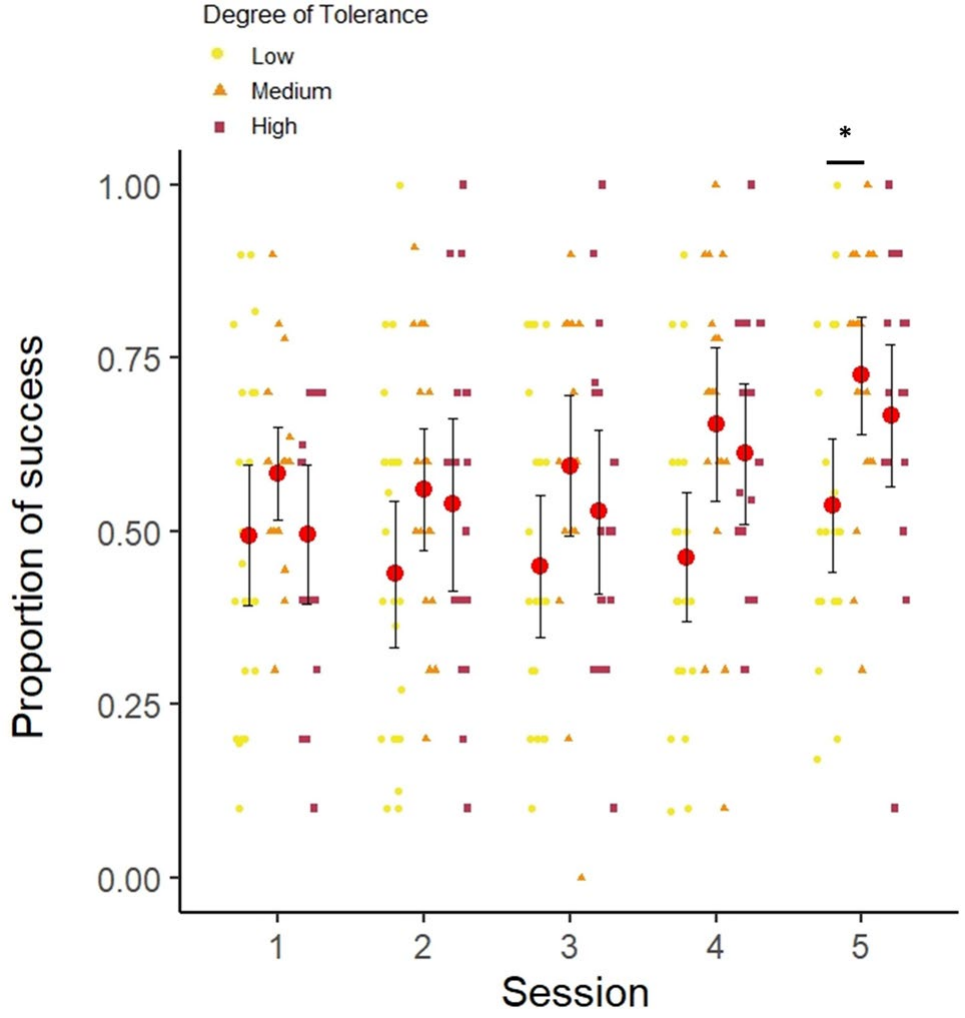
Mean proportion of success in a No-go trial in the Go/No-go task for each session in low, medium and high tolerance species. 95% CI are represented, red dots represent the mean per species, the mean for each individual is also represented: yellow dot (low tolerance species), orange triangle (medium tolerance species), purple square (high tolerance species). *P < 0.05 (from the Tukey Post Hoc test) (color figure online)

As the explanatory factor session had a main significant effect on the proportion of success on a “No-go” trial (*χ*^2^_1_ = 18.672, *N* = 64, *P* < 0.001, see Fig. 3 and Online Resource S13), with better performances as the number of sessions increased, we decide to look at subjects’ performances at their last session. We demonstrated an effect of tolerance on the proportion of success on a “No-go” trial at the last session (*χ*^2^_1_ = 9.683, *N* = 64, *P* < 0.01). We found that the species with intermediate and high levels of tolerance had higher scores than low tolerance species (see Fig. 3 and Online Resource S14). From the Tukey Post Hoc test (see Online Resource S14) we only found that the medium tolerance species had higher scores than the low tolerance species at the last session (*Z* = 2.854, *P* < 0.05). Therefore, we considered it as the true significant difference. The factors age, rank and sex did not have a main general effect on subject’s performances but the subjects in general improved their performances through sessions (see Online Resource S14). We also found that the low tolerance species performed below chance level for the last session (Wilcoxon test, *V* = 80.5, *P* = 0.532; *M* = 0.538, S.D. = 0.217). The medium tolerance species (Wilcoxon test, *V* = 179.5, *P* < 0.001; *M* = 0.725, S.D. = 0.180) and the high tolerance species both performed above chance level (Wilcoxon test, *V* = 134.5, *P* < 0.01; *M* = 0.667, S.D. = 0.206). Overall, the low tolerance species had lower performance in this Go/No-go task compared to the medium and high tolerance species which demonstrated a worse control of an action (see Table 2 for a summary of the results).

One macaque was not willing to participate in further testing after this task.

### Results for the *inhibition of a cognitive set*: Reversal learning task

**Fig. 4:**
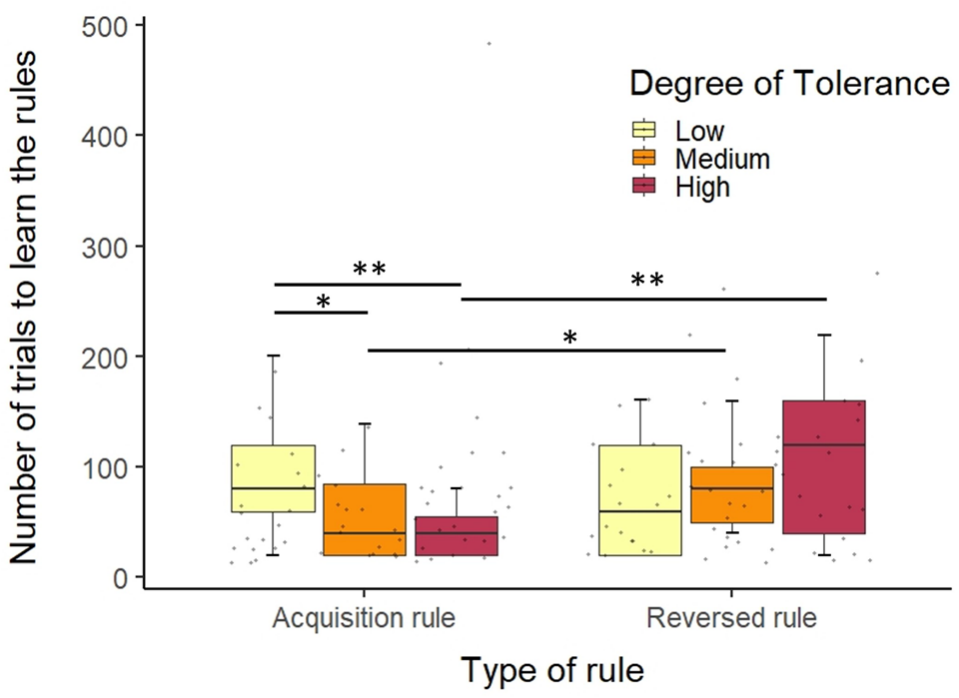
Number of trials to learn the rules (acquisition or reversed rules for each tolerance degree (Low, medium and high tolerance species). Low tolerance species needed more trials to learn the acquisition rule. Horizontal lines represent the 25th, 50th and 75th percentile and whiskers extend to 1.5 times the interquartile range. Black dots represent the mean for each individual. *P < 0.05, **P < 0.01 (from the pairwise comparison and the model comparisons) (color figure online)

For the acquisition rule, there was a significant main effect of the tolerance degree on the number of trials to learn the rule (*χ*^2^_2_ = 12.856, *N* = 63, *P* < 0.01). From the pairwise comparison, there was a significant difference between less and high tolerance species (*P* < 0.01) and between intermediate and low tolerance species (*P* < 0.05). Low tolerance species needed more trials to learn the first rule (*M* = 106.4, S.D. = 94.47) than medium tolerance species (*M* = 58, S.D. = 41.5) and high tolerance species (*M* = 40, S.D. = 20.58). For the reversed rule, there was no significant main effect on the tolerance on the number of trials to learn the rule (*χ*^2^_2_ = 1.038, *N* = 63, *P* = 0.361). From the Tukey’s post Hoc test, we found the same absence of difference between species for the reversed rule (see Online Resource S15). Low tolerance species needed as many trials (*M* = 81.67, S.D. = 76.2) to learn the rules as medium (*M* = 93.68, S.D. = 69.92) and high tolerance species (*M* = 117.78, S.D. = 96.5). Only medium (*χ*^2^_1_ = 4.2036, *N* = 18, *P* < 0.05) and high tolerance species (*χ*^2^_1_ = 10.238, *N* = 18, *P* < 0.01) needed more trial to learn the reversed rule compared to the acquisition rule (see Fig. 4 and Online Resource S15). Overall we demonstrated that the low tolerance species needed more trials to learn the acquisition rule compared to the medium tolerance species and the high tolerance species. All species needed the same number of trials to learn the reversed rule. The medium and high tolerance species needed more trials to learn the reversed rule compared to the acquisition rule (see Table 2). A higher number of trials required to learn the reversed rule would indicate a lower control of the interference effect of a previously learnt rule.

## Discussion

In line with our hypothesis, we demonstrated overall the best control of a distraction and emotions in the highly tolerant species and the worst control of distraction, emotions and actions in the less tolerant species. The performance of the medium tolerance species was intermediate with a good *inhibition of an action* and *a distraction* even though this species displayed few behavioural reactions toward pictures. Lastly when looking at the *inhibition of a cognitive set*, the medium and high tolerance species had better learning skills compared to the low tolerance species.

We found poor inhibitory control performances in the low tolerance species except in the *inhibition of a cognitive set*. First, in the *inhibition of a distraction* task, an increased emotivity and distractibility in this species, compared to the higher tolerant species, could be due to a stronger negative valence associated with social stimuli and particularly threatening conspecific faces. It is possible that, in this species the “open mouth threat” has a stronger negative valence compared to the other species and particularly compared to the more tolerant species. Interestingly, it has been demonstrated that the “silent bared-teeth”, has a different meaning depending on the species considered. This facial expression is used to express submission in rhesus and long-tailed macaques (Thierry 2007; Thierry et al. 2004) and in Tonkean macaques, the ‘silent bared-teeth’ is used to signal peaceful intentions and affiliation (Thierry et al. 1989). There is no formal signal of submission in Tonkean macaques (Thierry et al. 1989). Thus, in our study, this difference in the meaning of facial expressions, could explain why high tolerance species did not visibly react to the picture of conspecifics, as the meaning and valence of the facial expression displayed vary between species. Besides, it is possible that the threshold of arousal is higher in the most tolerant species and the design we used (using only pictures) was not powerful enough to elicit a visible behavioural response in this species. It would be interesting to compare performance of these three species but using positive stimuli (food or positive facial expression such as lipsmack). A study in Japanese macaques and chimpanzees showed that positively valenced stimuli had one of the most distracting effects (compared to neutral and threatening stimuli; Hopper et al. 2021). We could also replicate the same experiment using videos instead of pictures as animals have been shown to react strongly to videos (Fagot et al. 2010). One study in rhesus macaques using videos elicited spontaneous social behaviours such as gaze following and reciprocal facial expression, which was not previously observed using still pictures (Mosher et al. 2011). It would also be interesting to compare intra-species individual variations as we can see in Fig. 2 that low tolerance species seem to demonstrate more individual differences.

In the *inhibition of an action* task and in the *inhibition of a cognitive set* task, the low tolerance species also seem to be more impulsive as they had more difficulties in learning the task’s rule compared to the medium and high tolerance species. It is possible that the enhanced reactivity and impulsivity in this low tolerant species could impair their capability of focusing their attention in learning a new rule. For instance, in marmosets (*Callithrix jacchus*), high emotional reactivity impaired animals’ attention in cognitive testing (Schubiger et al. 2015). Furthermore, in the *inhibition of a cognitive set* task, the previously learnt rule only distracted medium and high tolerance species in learning a new rule. This could indicate a lower inhibition of a previously learnt rule. However, it is also possible that only the medium and high tolerance species truly comprehend and memorise the first acquisition rule which would explain why they had trouble reversing it. The low tolerance species could have learnt both rules by chance (they needed a high number of trials to learn both rules), without truly understanding it. However, this reversal learning task has been criticised for not truly measuring inhibitory control. Instead, it has been suggested that this task could be a measurement of cognitive flexibility (Izquierdo et al. 2017), which could also be crucial in navigating a complex social world. We also demonstrated in a previous study (Loyant et al. 2022), that rhesus macaques did not have consistent performance in the *inhibition of a cognitive set* task compared to the *inhibition of a distraction* task and the *inhibition of an action* task, showing a lack of contextual validity. It was also unclear if this task really elicited a prepotent response in the low tolerance species. Further studies comparing for instance cognitive flexibility abilities and reversal learning performances could try to disentangle the implication of each cognitive process in this task.

Finally, contrary to what we expected, in the *inhibition of an action* task, the high tolerance species did not have the best accuracy in this task (but only a tendency to be better than the low tolerance species). It is possible that the high tolerance species had the same inhibitory control skills as intermediate tolerant species in this task. This lack of difference could also be due to a high number of highly ranked males in our sample of high tolerance species which could have decreased the overall performance in this species. Male rhesus macaques have been shown to be more impulsive (Loyant et al. 2021). Similarly, human studies demonstrated that women outperform men on the no-go trials, indicating greater inhibition (Sjoberg and Cole 2018). It is possible that male performances slightly lowered the overall high tolerance species’ performances and thus decreased the difference between high and low tolerance species, with only a tendency to have significantly different performances.

Overall, we found that the more tolerant species had better inhibitory control skills than the less tolerant species in a battery of tests. Evolving in a more tolerant social group, considered socially more complex (Rebout et al. 2021), may be associated with better inhibitory control skills, corroborating the social intelligence hypothesis.

This relationship between social tolerance and cognitive skills was also demonstrated in social and physical cognitive tasks. In the social domain, several studies demonstrate that social tolerance is associated with better socio-cognitive performances. For instance in the pointing cup task, which involved cooperating with a human experimenter, more tolerant macaque species outperformed the less tolerant ones (Joly et al. 2017). In another cooperative task (simultaneously lifting a heavy stone), high tolerant macaque species (*Tonkean macaques*) performed better than low tolerance species (rhesus macaques; Petit et al. 1992). Similar findings were found in non-human primates when taking “co-feeding” (to allow close proximity of others while feeding) as a measurement of social tolerance (DeTroy et al. 2021). For instance, bonobos, considered a more tolerant species, were better in a cooperative task than the less tolerant chimpanzees (Hare et al. 2007). More generally in the Primate Cognition Task Battery, bonobos were more skilled in social tasks (theory of mind task or social causality task) than the less tolerant chimpanzees (Herrmann et al. 2010).

The relationship between social tolerance and physical cognition is less clear. For instance, a study demonstrated that bonnet macaques (*Macaca radiata*, a tolerant macaque species placed on grade 3 on the scale of social tolerance (Thierry 2007; Thierry et al. 2004), outperformed rhesus macaques on spatial short memory task and on an object-reward association task (Comrie et al. 2018). These results should be interpreted with caution as only females were tested in bonnet macaques and mostly male in rhesus macaques (24 males out of 35 individuals). This unbalanced sampling could have led to a sex bias in favour of bonnet macaques (Loyant et al. 2021). Harrison et al. (2021) also demonstrated that a more tolerant group of chimpanzees (measured by co-feeding and socio-positive interactions) had better flexibility skills in tasks of foraging (subjects needed to use different types of tools depending on the context) compared to a less tolerant group. Contrarily, in another study, chimpanzees, considered as a low tolerance species, had better performances in the use of tools and the understanding of physical causality than bonobos, a more tolerant species (Herrmann et al. 2010). Further studies are needed to better understand the impact of social tolerance on performances in social or non-social tasks.

In our study, we did not find a linear correlation between social tolerance and inhibitory control skills as the picture was less clear for the species with medium degree of social tolerance. Medium tolerance species had, overall, good control of their impulsions compared to low tolerance species and high tolerance species (for only one measurement of the *inhibition of action*). However this medium tolerance species was still demonstrating emotionality levels similar to low tolerance species. It is possible that fitting species with intermediate levels of social tolerance in the four-grade scale might not be as straightforward. Thierry (2007) calls for caution as classifying species along a discrete and bipolar scale is inevitably reducing, particularly for species with intermediate level of tolerance. The author states that each species should be represented as a cluster of points (representing each population studied) along a continuous scale. In addition, the phylogenetic model of macaque social tolerance is based on a series of studies focused on female behaviours (Thierry 2007; Thierry et al. 2004). As we demonstrated in (Loyant et al. 2021), male and female macaques can drastically differ in their behaviours, it is thus possible that a new phylogenetic model based only on males’ behaviours, could lead to unexpected findings. In this line, several authors suggested that social tolerance can also take root in another systematic variation model: the socio-ecological model of female relationships (Isbell and Young 2002; Sterck et al. 1997). There is a consensus that social organisation patterns in behaviours in primates are linked to the environment in which they have evolved (Isbell and Young 2002; Sterck et al. 1997). According to this model female relationships can be explained by a combination of variables such as predator vulnerability, food distribution, population density and inter- and intra-group competition. DeTroy et al. (2022) suggest that the socio-ecological model can be represented as an inverted U-shape: “both a lack of dominance hierarchy (i.e. egalitarianism) and high levels of despotism prohibits social tolerance, while an intermediate level of despotism, combined with the reliance of dominant individuals on coalitionary support foster social tolerance”. It could be possible that our lack of clear relationship between social tolerance and inhibitory control skills could be due to the characterization of social tolerance. According to the socio ecological model, the medium tolerance species could be, in fact, more tolerant than the species with the higher degree of social tolerance (given by Thierry’s classification; Thierry 2007; Thierry et al. 2004). More studies are needed to clarify the definition and measurement of social tolerance but also to better understand the relationship between social complexity and the evolution of socio cognitive skills. One approach to reinforce our findings could be to compare inhibitory control skills in species with intermediate degree of social tolerance (e.g., Barbary macaques, grade 3 on Thierry’s classification) to the performances of our sample of macaque species.

Frequently splitting and merging in subgroups of variable composition (fission–fusion dynamics) has also been proposed as one other aspect of social complexity influencing inhibitory control (Amici et al. 2008). A primate comparative study presented 5 tasks putatively measuring inhibitory control (the A-not B task, a variant of the detour reaching tasks, a middle cup task and a measure of self-control) to 7 species of non-human primates (Amici et al. 2008). The authors found an association between performances on these tasks and the social structure of these species. Species living in more dynamic and fluid social environments (fission–fusion societies) outperformed those having more cohesive group structures. The authors concluded primates living in more complex social groups often require inhibition of inappropriate prepotent responses in a dynamic social environment, and this partly explains why they performed better in Detour tasks. It would be interesting to replicate these results by comparing inhibitory control skills, thanks to our battery of tasks, in species differing in fission–fusion dynamics. In addition, it is assumed that macaques are all cohesive societies but there might be differences in their fusion-fission dynamic. For instance, within-group competition may result in different patterns of group fission with variation between species (Thierry et al. 2004). It would be interesting to investigate further the relation between macaque fission–fusion dynamics and inhibitory control.

In the literature, there are still some debates between the supporters of the social model versus the defenders of the ecological model especially when looking at the selective forces that favour the evolution of cognition (Amici et al. 2008; MacLean et al. 2014). We do not consider them mutually exclusive. The species tested in this study might also face different ecological challenges, such as predation risk for instance, which could shape their inhibitory control skills. Long-tailed macaques are smaller than the other two species, primarily arboreal, they live along rivers and in forest margins occupied by numerous predators (Crockett and Wilson 1980; Fooden 2006). These factors could have shaped them to be more reactive and cautious to their environment (thus being more emotive as seen in our results). The larger-bodied Tonkean macaques face far less predators on the island of Sulawesi (Whitten and Henderson 2012). This lesser risk of predation could select quieter and less reactive behaviours (thus being less emotive and impulsive as in our results). Rhesus macaques favour open habitats where they are likely to encounter numerous smaller predators (Thierry et al. 2004) and thus may benefit from being highly reactive and defending themselves by aggressive confrontation (thus being more impulsive and reactive as in our results). Species’ inhibitory control (which could be associated with aggressivity, emotionality and impulsivity) might therefore be adaptive to ecological pressure as well.

Our study suffers from several limitations common in primate studies (for review see ManyPrimates 2019). First, although very reasonable for this kind of study, our sample size was limited, which could have decreased the power of our analysis. In further research it would be interesting to use collaborative projects such as ManyPrimates to increase the number of subjects and to iron out differences between groups (ManyPrimates 2019). Then, the tested subjects did not have the same experience with cognitive experiment. The large group of highly tolerant access had ad libitum access to touch screen modules with cognitive experiments (e.g., delay match-to-sample task) which could explain their good performances in our battery of tasks. However, the medium tolerance species, which did not have any experience of any type of cognitive testing, were better than the high tolerance species in the Go/No-go task. Furthermore, four individuals from the high tolerance species never worked with pictures before and showed no difference in performances compared to the group that previously worked with neutral conspecific faces. Thirteen of the low tolerance species from the MRC already took part in an experiment in which they had to look at pictures, but they were still highly reactive to pictures of conspecifics. Moreover, low tolerance species, from two different institutions, performed similarly poorly in each of the three inhibitory control tasks. Rhesus macaques demonstrated no significant difference in performances between institutions in the Distraction task: individuals were similarly highly reactive to stimuli. Besides, rhesus macaques from both institutions had similarly low accuracy in the Go/No-go task and they also needed a higher number of trials to learn the Reversal learning task (see Online Resource S6). Thus, previous experience could not totally account for the differences in inhibitory control we found.

Similarly, the high tolerance species were semi-free ranging, housed in large wooded enclosures. Differences in captive conditions could explain differences in cognitive abilities as a more enriched environment could help the individuals to develop better cognitive capacities (Schubiger et al. 2020). For instance, shelter dogs displayed poorer performances in the A-not-B task than pet dogs (Fagnani et al. 2016). According to the authors, shelter dogs might live in an impoverished environment with less interaction with humans which would decrease their chances to learn to inhibit certain behaviours. However, high tolerance species were still at the same level of performance than the medium tolerance species for the Go/No-go and the Reversal learning task, so the environment did not lead to a difference in cognitive abilities.

Altogether, we demonstrated that low tolerance species have lower inhibitory control than other more tolerant species. But the findings do not follow a linear increase of inhibitory control performances from low to intermediate and high tolerance, most probably reflecting a more diverse social complexity within the genus than previously acknowledged. More comparative research is needed to have a better understanding of the selective pressures driving the evolution of inhibitory control.

## Supplementary Information

Below is the link to the electronic supplementary material.

Supplementary file1 (DOCX 3382 KB)

## Data Availability

The Online Resource, MATLAB codes, stimuli for every task, the datasets generated during the current study, are stored in Github: https://github.com/Psychology-inhibitory-control/INHIBITORY-CONTROL-AND-SOCIAL-TOLERANCE.git.
