# Supplementary material for: Tolerant macaque species are less impulsive and reactive
Source: Anim Cogn. 2023 May 28;26(5):1453–66. doi: 10.1007/s10071-023-01789-8 (PMC10442267; doi:10.1007/s10071-023-01789-8)
Supplement: Supplementary file 1 — Supplementary file1 (DOCX 3382 KB) [file 10071_2023_1789_MOESM1_ESM.docx]

# Online Resource Animal Cognition

**Tolerant macaque species are less impulsive and reactive**

Louise Loyant^1^, Bridget M. Waller^2^, Jérôme Micheletta^1^, Hélène Meunier^3^, Sébastien Ballesta^3^, and Marine Joly^1^

^1^ Centre for Comparative and Evolutionary Psychology, Department of Psychology, University of Portsmouth, Portsmouth, United Kingdom

^2^ Department of Psychology, Nottingham Trent University, Nottingham, United Kingdom

^3^ UMR (7364), Laboratoire de Neurosciences Cognitives et Adaptatives, CNRS and Université de Strasbourg, Strasbourg, France

Corresponding Author:

Louise Loyant^1^

Department of Psychology, King Henry I Street, King Henry Building, Portsmouth PO1 2DY, United Kingdom

Email address: louise.loyant@port.ac.uk

# S1 Training procedures

***Training phase 1***

Monkeys were trained by positive reinforcement (based on food rewards) to touch a red square target (10 x 12 cm). The target was moved in all directions and the monkey should follow it for at least 20 trials in a row.

***Training phase 2***

The second training phase used a rectangular transparent Plexiglas sheet (42 x 30 cm) with a plastic red square target (10 x 12 cm) moved by hand. Monkeys were progressively rewarded for approaching the Plexiglas sheet, touching the target as it moved. This phase was judged as complete if the macaques followed the target 20 times in a row.

***Training phase 3***

After completion of the first training phase, the touchscreen was introduced. The aim of training phase 2 was to be trained to touch a target on the screen.

**Stimuli**

The target was a red (RGB 255, 0, 0) rectangle of a maximum of 1200 x 1000 pixels (ca. 32 x 26 cm) and was gradually reduced to 360 x 500 pixels (ca. 10 x 13 cm).

**Procedure**

When the program was launched, the experimenter entered the name of the individual, the number of trials per session, the size of the target and the time limit to complete the task. Every session was initiated by the subject touching a red cross located in the centre of the screen, starting the time recording. Then, the target was displayed in the middle of a white background. Once the subject touched the target, a high-pitched chime (composed of 3 sound frequencies: 800, 1300 and 2000 Hz) was played, the timer was stopped, and the reward was given. After an inter-trial of 2000 ms, with only the white background displayed, the next trial was presented. Based on a pilot study (N = 4 subjects), we set up a maximum response time of 35s.

If the subject left the testing area or was not focusing attention on the screen the session was aborted. If the target was not touched within the time limit (35 s), the timer was paused, a red cross appeared in the centre of the screen until the session was resumed by touching it. The size of the target was gradually reduced to 360 x 500 pixels (ca. 10 x 13 cm).

Once the macaques successfully performed 20 times in a row with the target in the centre of the screen, the target was displayed randomly at the far left or right of the screen. This phase was judged as complete if the macaque followed the target 20 trials in a row. If the subject stayed inactive for more than 5 min the session was stopped and continued the next testing day, if the subject did not participate for three testing days in a row the subject was excluded from the task.

# S2 Rank calculation

We considered high ranking individuals the two subjects at the top of the hierarchy at the time of the testing (e.g., in mixed groups, the top-ranking male and the top-ranking female).

### ***Rank calculation at the MRC-CFM***

**Calculated ranks by David’s Scores:**

Ranks obtained from David’s Scores (DS) (De Vries et al. 2006) were calculated in 2016. They were also based on agonistic interactions recorded *ad libidum*. Agonistic behaviours included threats (e.g., open mouth threat), displacements (i.e., a macaque approaches another who departs immediately), chases, and physical conflict (e.g., bite, slaps). These calculated ranks were regularly monitored and updated during observations and training sessions by caretakers. After a regression analysis all calculated ranks were found significant.

Total Number of conflicts: 734

The number of conflicts recorded in each group ranged from 48 to 136 conflicts.

The number of observations recorded for each group ranged from 4 to 14 observations.

**Observed ranks:**

To confirm the calculated ranks at the time our experiments were conducted we coded agonistic behaviour with a focus on the studied subject. A blind observer recorded agonistic interactions (winner/loser) between a focal individual and other members of the group during the training and test sessions. Agonistic behaviours included threats (e.g., open mouth threat), displacements (i.e., a macaque approaches another who departs immediately), chases, and physical conflict (e.g., bite, slaps). If the male never lost, he was considered as high ranked. If the female never lost against other females, she was considered as high ranked (H), otherwise she was considered low ranked (L).

| **Females** | **loser (suppl/threat)** | **winner (suppl/threat)** | **No observations** | **Sex** | **Observed rank** | **Rank from DS** |
| --- | --- | --- | --- | --- | --- | --- |
| Ylang | 18 | 30 | 26 | F | L | L |
| Adele | 32 | 13 | 23 | F | L | L |
| Rach | 0 | 21 | 32 | F | H | H |
| Ybbi | 0 | 8 | 18 | F | H | H |
| Blossom | 0 | 33 | 27 | F | H | H |
| Tes | 1 | 7 | 12 | F | L | L |
| Aqua | 4 | 5 | 10 | F | L | L |
| Saphy | 0 | 8 | 7 | F | H | H |
| Talula | 0 | 34 | 27 | F | H | H |
| **Males** | **loser (suppl/threat)** | **winner (suppl/threat)** | **No observations** | **Sex** | **Observed rank** | **Rank from DS** |
| Yew | 0 | 6 | 22 | M | H | H |
| Yorkie | 0 | 34 | 39 | M | H | H |
| Yosser | 0 | 3 | 40 | M | H | H |
| Zazy | 0 | 2 | 36 | M | H | H |
| Sol | 0 | 81 | 19 | M | H | H |
| Vincent | 0 | 16 | 26 | M | H | H |
| Abbott | 0 | 14 | 28 | M | H | H |
| Sequel | 0 | 14 | 22 | M | H | H |
| Nodon | 0 | 41 | 31 | M | H | H |
| Star | 0 | 23 | 29 | M | H | H |
| Plum | 0 | 31 | 18 | M | H | H |
| Yeti | 5 | 0 | 15 | M | L | L |

### ***Rank calculation at the CdP:***


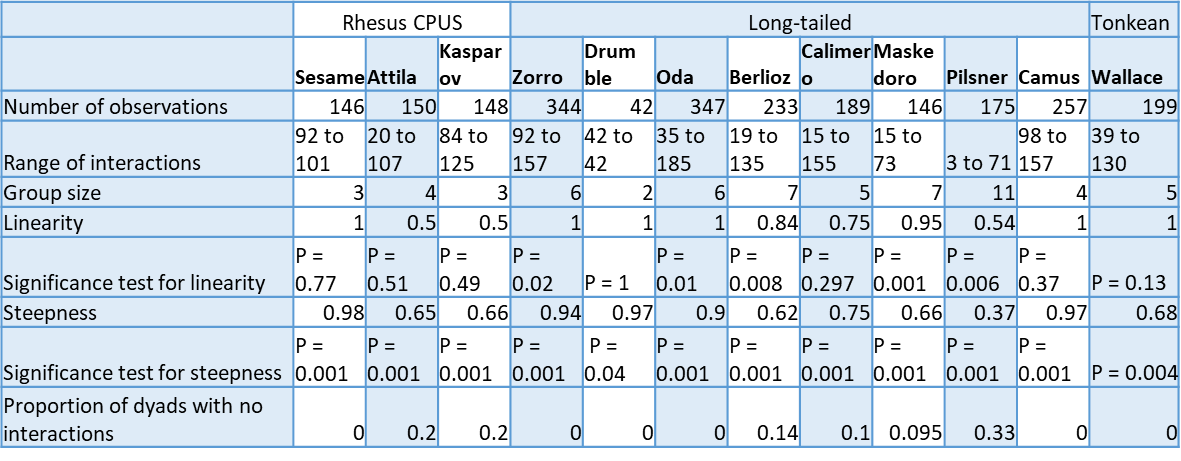
Rank of the subjects were also based on agonistic interactions recorded *ad libidum* using David’s score and Elo-ratings (a ranking method developed by Elo 1978, see also Neumann et al. 2011). Agonistic behaviours included threats (e.g., open mouth threat), displacements (i.e. a macaque approaches another who departs immediately), chases, and physical conflict (e.g. bite, slaps). Here is presented the number of observations per group, the range of interactions, the group size, the coefficient of linearity and associated P value, the coefficient of steepness and associated P value and the proportion of dyads with no interactions. We considered high ranking individuals the two subjects at the top of the hierarchy at the time of the testing (e.g. the top-ranking male and the top-ranking female in mixed groups).

The rank of the individuals from the large group of Tonkean macaques was automatically calculated in real-time using the modules installed in their enclosure. Ballesta and colleagues (2020), demonstrated that the calculation of the ranking they developed, using displacements occurring in the modules, was highly correlated with traditional behavioural observations (using agonist interactions as we did).

# S3 Design of the Distraction task

Every session was initiated by the subject touching a red cross in the centre of the screen. Then the timer started, and the subject had to touch a target (a red rectangle of 10 x 13 cm) randomly displayed at the far left or right of the screen. When the subject successfully touched the target, a high-pitched chime was played, the timer was stopped, and the reward was given. After an inter-trial of 2000 ms with only a white background displayed, the next trial was presented. Such a trial without a distractor was considered as a “Control” trial. Two “Control” trials were followed by a block of four trials with pictures from the same categories (either four pictures of objects, neutral or threatening conspecific faces). Each block of pictures of the same category was seen 2 times per session. The distractors were displayed at the centre of the screen at the same time as the regular target. We used 3 categories of stimuli. The category “Object” included a leather ball, leather bag, brown stone and wooden log (see below).


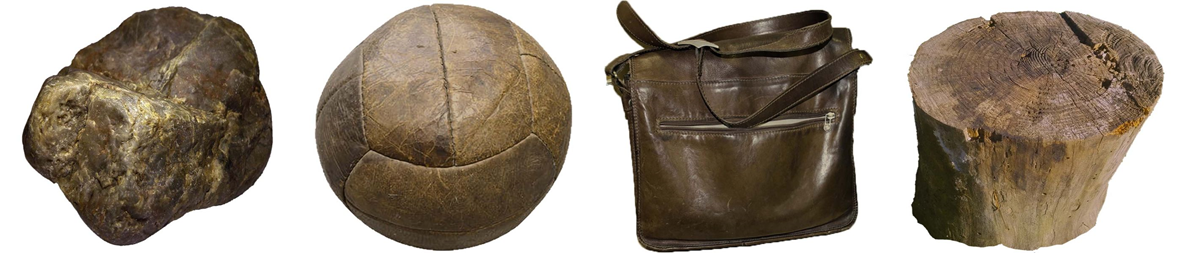


*“Object trials”. Objects presented in the Distraction task (“Object” trials). We used a rock, a leather ball, a leather bag and a log of wood as stimuli.*

For each species, the conspecific pictures were also chosen to be as realistic as possible, depicting a frontal view of the face and the torso of four unknown adult macaques. The “Neutral” conspecific included, for each species, four pictures of individuals with a neutral facial expression. The “Threatening” conspecific also included for each species, four pictures showing a “open mouth threat” facial expression (see below).

*Low tolerance species:*


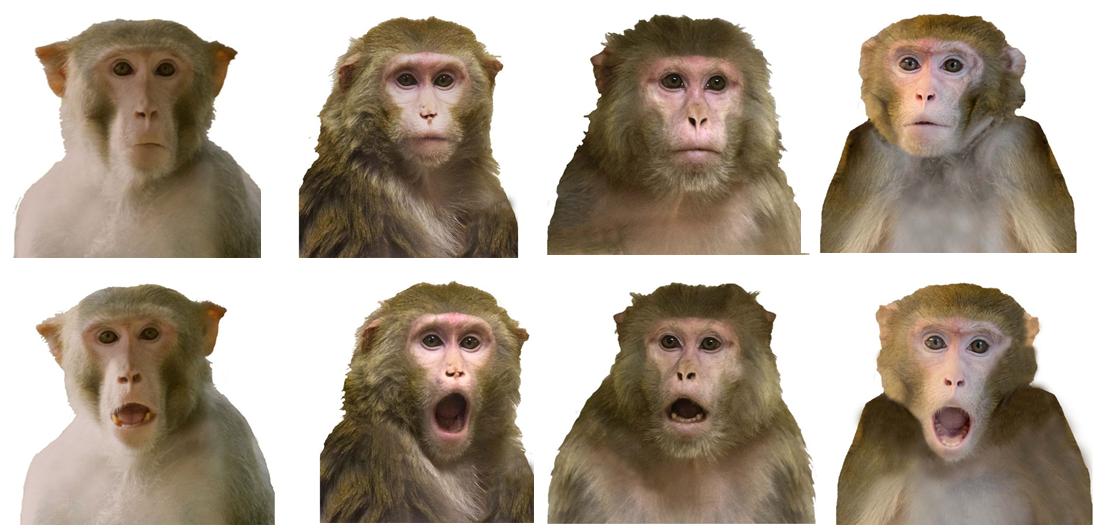


*Medium tolerance species:*


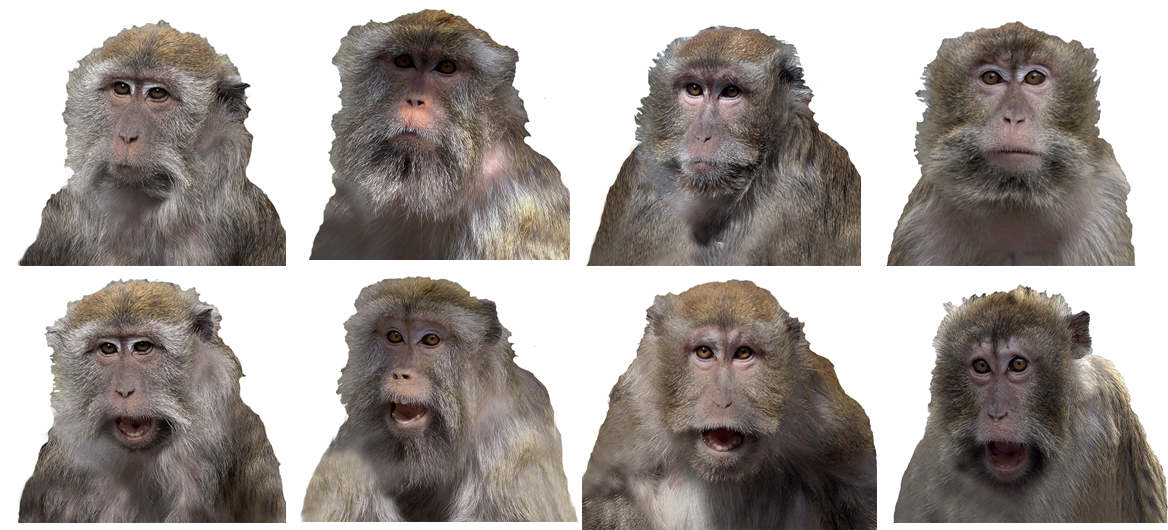


*High tolerance species*

*
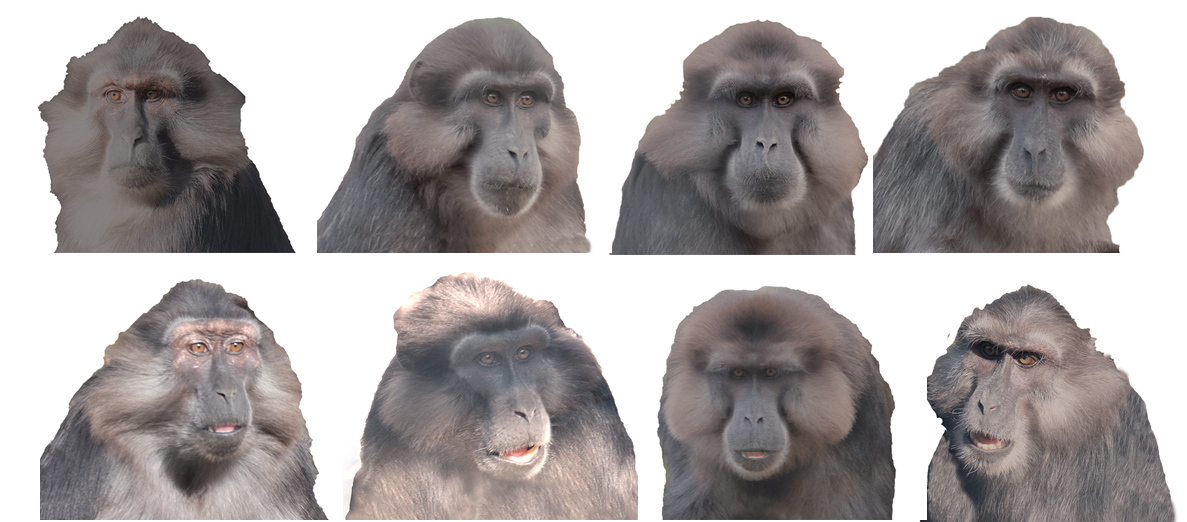
“Neutral” and “Threatening” trials. Stimuli used in the Distraction task in rhesus macaques. The first row displays neutral unknown conspecific faces (“Neutral” trials). The second row displays the same conspecifics but with a threatening facial expression (“Threat” trials).*

Each subject was tested in 3 sessions of 36 trials. The subjects were not rewarded to touch the distractors and the screen remained the same until the target on the side was touched. During a pilot study (N = 4 subjects, these data were not included in our analysis), we observed that the subjects were, for a long duration, intensely reacting to the pictures of their conspecifics’ faces (lip-smacking, stares and threats) so we set up a maximum response time of 35 s (at the condition that the subject kept looking at the screen). This time period allowed the subject to display a behavioural response, overcome it, and continue the task. If the subject did not touch the target within 35 sec, the response latency was not taken into account in the analysis and the red cross appeared. Each block and trial were counterbalanced across subjects.

# S4 Design of the Go/No-go task

In the Go/No-go task, the subjects need to respond to frequently presented stimuli while withholding prepotent response to infrequently presented no-go stimuli. Each subject was tested in 5 sessions of 40 trials. A “Go” (red rectangle of 16x18 cm) or a “No-go” stimulus (green circle of 16x16 cm) appeared randomly in the centre of the screen. The “Go” stimulus was preceded by a high-pitched sound (0.6 sec before the stimulus appeared) and the “No-go” stimulus a low pitch sound to help the subjects to anticipate the next trial. The “Go” stimuli appeared 75% of the 40 trials to elicit a prepotent response toward the screen. If the screen was touched outside the stimulus no sound was produced and the trial continued. The “Go” stimulus stayed on the screen until it was touched. We set up a maximum response time (i.e., touching the “Go” stimuli) of 15 s after this the red cross appeared on the screen and the response latency was not recorded. From a pilot study conducted with a shorter response limit, we observed that the subjects frequently left the testing session as they were not rewarded on each “Go” trial. The “No-go” stimulus disappeared if not touched during 2000 ms and the subject received a reward. If the “No-go” stimulus was touched during this lapse of time, a blank white background appeared for 3000 ms (as a time out), an “incorrect” sound (with frequency 800, 1300, 2000 Hz) was produced and the reward was not given.

# S5 Design of the Reversal learning task

At the beginning of the task, two stimuli, a “Go” rewarded stimulus (a red square of 15.34 x 15.34 cm) and a “No-go” unrewarded stimulus (a green circle of 15.34 cm of diameter), were displayed at the same time on the screen at counterbalanced locations (left or right of the screen). When the subject touched the “Go” stimulus, the usual “correct” sound was played, the subject received a reward, and a new trial began. If the subject touched the incorrect stimulus the “incorrect” sound was played, the subject did not receive a reward and the two stimuli stayed on the screen until the correct stimulus was touched. If the background was touched nothing happened. We set up a maximum response time (i.e. touching the “Go” stimuli) of 15 s after this the red cross appeared on the screen and the response variables were not recorded. We set up this response limit to keep the subjects engaged with the task. From a pilot study we observed that this period of time allowed the subject to frequently receive a reward and to keep engaged with the task. A session consisted of 40 trials. Once a criterion of success was achieved (75% of correct trials out of 20 trials, i.e., the subjects touched the correct stimulus from the first attempt), the rule was reversed: the correct stimulus became the incorrect and the incorrect the correct. One male macaque was excluded from the study as he did not reach the first criterion. The reversed session was continued until the success criterion was reached again (75% of success for the whole session).

# S6 Effect of the factor institution on the inhibitory control scores from the model comparison

For the distraction task, individuals from the low tolerance species came from two institutions but there was no difference in their Distraction control score (χ^2^ _1_ = 0.292, N = 28, P = 0.589). For the Go/No-go task, individuals from the low tolerance species came from two institutions but there was no difference in their proportion of success in this task (χ^2^ _1_ = 0.292, N = 28, P = 0.589). For the Reversal learning task, the model with the factor institution did not have a main significant effect when compared with the null model (χ^2^_1_ = 01.878, N = 28, P = 0.171). Therefore we pooled all of the individuals from the low tolerance species together for each score.

# S7 Comparison of response latency between the three species

| **Response latency** | **Estimate** | **Std. Error** | **t value** | **Pr(>\|t\|)** |
| --- | --- | --- | --- | --- |
| (Intercept) | 4732.576 | 569.781 | 8.305 | 1.47E-12 |
| age | 89.058 | 34.663 | 2.569 | 0.0123 |
| Sex (male) | 502.453 | 362.173 | 1.387 | 0.1709 |
| Rank (high) | 352.350 | 395.550 | 0.891 | 0.3763 |
| Tolerance (low) | -593.691 | 456.831 | -1.299 | 0.198 |
| Tolerance (medium) | 818.972 | 466.010 | 1.757 | 0.0836 |
| trial | -10.153 | 10.450 | -0.971 | 0.331 |
| session | -561.78 | 98.2642 | -5.7170 | 1.21E-08** |

*Results of the LMM for the response latency in the training trials (touching a target on the screen, phase 2 of the training, 20 times in a row). Explanatory variables were individual characteristics (sex, age and tolerance), experimental determinants (trial and session). All full models the individual ID as a random factor. The Estimates (representing the change in the dependent variable relative to the baseline category of each predictor variable), Standard Error, t-value and p-value are represented. 6822 observations were analysed. * p < 0.05, ** p < 0.01, *** p < 0.001.*

The factor tolerance did not influence the response latency of the subjects in the last step of the training sessions (χ^2^ _1_ = 12.69, N = 66, P > 0.05).

# S8 Distraction control score for all sessions together

| **Predictors** | | **Estimate** | **Std. Error** | **t value** | **Pr(>\|t\|)** |
| --- | --- | --- | --- | --- | --- |
| **(Intercept)** | | 95.019 | 2.157 | 44.042 | 0.000 |
| **age** |  | -0.012 | 0.132 | -0.103 | 0.9127 |
| **sex** | female vs male | 0.502 | 1.360 | 0.369 | 0.669 |
| **tolerance** | low vs high | 2.608 | 1.709 | 1.497 | 0.091 |
|  | high vs medium | 3.349 | 1.736 | -1.929 | 0.049* |
|  | low vs medium | 5.922 | 1.560 | -3.797 | 0.000*** |
| **type of picture** | control vs picture | 0.553 | 0.164 | 3.382 | 0.001** |
|  | object vs face | 0.877 | 0.2777 | 3.162 | 0.002** |
|  | threat vs neutral | -1.194 | 0.482 | -2.475 | 0.013* |
| **trial** |  | -0.075 | 0.030 | -2.506 | 0.012* |
| **session** |  | -2.780 | 0.331 | -8.405 | 0.000*** |

*Results of the LMM for the normalised Distraction control score (Distraction task) when all the sessions were taken together. Explanatory variables were individual characteristics (sex, age and tolerance), experimental determinants (type of picture, trial and session). All full models included the type of picture nested in the individual ID as a random factor. The Estimates (representing the change in the dependent variable relative to the baseline category of each predictor variable), Standard Error, t-value and p-value are represented. 6822 observations were analysed. * p < 0.05, ** p < 0.01, *** p < 0.001.*

# S9 Results for the Distraction control score for session 1

| **Predictors** | | **Estimate** | **Std. Error** | **t value** | **Pr(>\|t\|)** |
| --- | --- | --- | --- | --- | --- |
| **(Intercept)** | | 96.243 | 3.352 | 28.708 | 0.000 |
| **age** |  | 0.044 | 0.237 | 0.187 | 0.853 |
| **sex** | male vs female | -0.909 | 2.352 | -0.386 | 0.701 |
| **tolerance** | low vs high | 7.535 | 2.847 | -2.647 | 0.010* |
|  | high vs medium | 0.282 | 3.133 | -0.09 | 0.929 |
|  | low vs medium | 7.817 | 2.923 | -2.675 | 0.009** |
| **type**  **of picture** | control vs picture | 1.245 | 0.301 | 4.136 | <0.000*** |
|  | object vs face | 1.332 | 0.494 | 2.694 | 0.0071** |
|  | threat vs neutral | -2.314 | 0.865 | -2.675 | 0.008** |
| **trial** |  | -0.021 | 0.056 | -0.375 | 0.708 |

*Results of the LMM for the normalised Distraction control score (Distraction task) for session 1. Explanatory variables were individual characteristics (sex, age and tolerance), experimental determinants (type of picture and trial). All full models included the type of picture nested in the individual ID as a random factor. The Estimates (representing the change in the dependent variable relative to the baseline category of each predictor variable), Standard Error, t-value and p-value are represented. 2102 observations were analysed. * p < 0.05, ** p< 0.01, *** p < 0.001.*

# S10 Results of the Tukey Post Hoc Tests of the Distraction control score for session 1

| **Tolerance degree** | **Estimates** | **Std. Error** | **Z value** | **Pr (>\|z\|)** |
| --- | --- | --- | --- | --- |
| low - high | 7.535 | 2.487 | -2.47 | 0.022* |
| high - medium | 0.282 | 3.133 | 0.090 | 0.996 |
| low - medium | 7.817 | 2.923 | -23.675 | 0.021* |

*Results of the Tukey HSD test comparing the normalised Distraction control score for each tolerance degree (low, medium and high tolerance species), for session 1. * p < 0.05*

S11 Results for the Tukey HSD test comparing the normalised Distraction control score for session 1

| **Type of picture** | **Estimates** | **Std. Error** | **z value** | **Pr (>\|z)** | |
| --- | --- | --- | --- | --- | --- |
| Control - Neutral | -3.995 | 1.552 | -2.573 | 0.049* |  |
| Object - Neutral | -1.678 | 1.706 | -0.984 | 0.758 |  |
| Threat - Neutral | 4.615 | 1.731 | 2.667 | 0.038* |  |
| Object - Control | 2.317 | 1.553 | 1.492 | 0.441 |  |
| Threat - Control | 8.61 | 1.579 | 5.452 | <0.001*** |  |
| Threat - Object | 6.293 | 0.729 | 3.64 | 0.002** |  |

*Each type of picture are presented: Control (no picture presented), Neutral (a picture of a neutral conspecific face presented), Threat (a picture of the face of a threatening conspecific is presented), Object (a picture of an object). * p < 0.05, ** p< 0.01, *** p < 0.001.*

# S12 Result for the number of behavioural reactions

|  | | **Tolerance** | | |
| --- | --- | --- | --- | --- |
|  |  | Low | Medium | High |
| **Type of pictures** | Control | 0 | 0 | 0 |
|  | Object | 0 | 0 | 0 |
|  | Neutral | 6 | 4 | 0 |
|  | Threat | 18 | 4 | 0 |

*Summary of the number of emotional reactions (threats, lips smacking and teeth chattering toward the screen) for each tolerance degree (low, medium and high tolerance species) and the type of picture (control: no picture, picture of an object, picture of a neutral conspecific and picture of a threatening conspecific).*

| **Tolerance degree** | **Estimates** | **Std. Error** | **Z value** | **Pr (>\|z\|)** |
| --- | --- | --- | --- | --- |
| high - low | -0.214 | 0.068 | 3.176 | 0.0043** |
| high - medium | -0.100 | 0.056 | 1.791 | 0.1725 |
| low - medium | 0.114 | 0.088 | 1.305 | 0.3923 |

*Result of the Tukey HSD test comparing the number of emotional responses for each tolerance degree (low, medium and high tolerance species) all sessions together. ** p < 0.01*

| **Type of picture** | **Estimates** | | **Std. Error** | | **z value** | | **Pr (>\|z)** |
| --- | --- | --- | --- | --- | --- | --- | --- |
| Control - Neutral | | -0.152 | | 0.061 | 2.492 | 0.0611 | |
| Object - Neutral | | -0.152 | | 0.061 | 2.492 | 0.0611 | |
| Threat - Neutral | | 0.182 | | 0.134 | -1.356 | 0.527 | |
| Object - Control | | 0.000 | | 7.03e-6 | 0.000 | 1.000 | |
| Threat - Control | | 0.333 | | 0.120 | -2.788 | 0.027* | |
| Threat - Object | | 0.333 | | 0.120 | -2.788 | 0.027* | |

*Results of the Tukey HSD test comparing the number of emotional reactions, for each type of picture: Control (no picture presented), Neutral (a picture of a neutral conspecific face presented), Threat (a picture of the face of a threatening conspecific is presented), Object (a picture of an object). * p < 0.05*

# S13 Results for the Go/No-go task, all sessions together

| **Predictors** |  | **Estimate** | **Std. Error** | **z value** | **Pr (>\|z\|)** |
| --- | --- | --- | --- | --- | --- |
| **(Intercept)** |  | 0.168 | 0.297 | 0.567 | 0.571 |
| **sex** | male vs female | -0.025 | 0.177 | -0.142 | 0.887 |
| **age** |  | -0.017 | 0.017 | -0.958 | 0.338 |
| **tolerance** | low vs high | -0.426 | 0.214 | -1.992 | 0.046* |
|  | medium vs high | 0.300 | 0.231 | 1.303 | 0.192 |
|  | medium vs low | 0.726 | 0.219 | 3.322 | 0.001** |
| **trial** |  | -0.001 | 0.003 | -0.453 | 0.650 |
| **session** |  | 0.116 | 0.027 | 4.311 | 0.000*** |

*Results of the GLMM for the probability of success in the Go/No-go task for a No-go stimulus when all the sessions were taken together. Explanatory variables were individual characteristics (sex, age and tolerance), experimental determinants (trial and session). All full models included the type of picture nested in the individual ID as a random factor. The Estimates (representing the change in the dependent variable relative to the baseline category of each predictor variable), Standard Error, t-value and p-value are represented. 3150 observations were analysed. * p < 0.05, ** p< 0.01, *** p < 0.001.*

# S14 Results for the Go/No-go task, last session

| **Predictors** |  | **Estimate** | **Std. Error** | **z value** | **Pr (>\|z\|)** |
| --- | --- | --- | --- | --- | --- |
| **(Intercept)** |  | 0.355 | 0.369 | 0.962 | 0.336 |
| **sex** | male vs female | -0.442 | 0.251 | -1.761 | 0.078. |
| **age** |  | -0.005 | 0.024 | -0.224 | 0.823 |
| **tolerance** | low - high | -0.701 | 0.301 | 2.334 | 0.020* |
|  | medium - high | 0.189 | 0.326 | 0.579 | 0.563 |
|  | medium - low | 0.890 | 0.312 | 2.854 | 0.004** |
| **trial** |  | 0.003 | 0.008 | 0.403 | 0.687 |

*Results of the GLMM for the probability of success in the Go/No-go task for a No-go stimulus for session 5. Explanatory variables were individual characteristics (sex, age and tolerance) and trial. All full models included the type of picture nested in the individual ID as a random factor. The Estimates (representing the change in the dependent variable relative to the baseline category of each predictor variable), Standard Error, t-value and p-value are represented. 624 observations were analysed. * p < 0.05, ** p< 0.01*

When looking at the Tukey Post Hoc test we found only the difference between the Low tolerance species and the medium tolerance species. Therefore, we considered it as the true significant difference.

| **Tolerance degree** | **Estimates** | **Std. Error** | **Z value** | **Pr (>\|z\|)** |
| --- | --- | --- | --- | --- |
| low - high | -0.702 | 0.301 | 2.334 | 0.051. |
| medium - high | 0.189 | 0.326 | -0.579 | 0.813 |
| medium - low | 0.890 | 0.312 | 2.854 | 0.012* |

*Results of the Tukey HSD test comparing the success on a No-go trial for each tolerance degree (low, medium and high tolerance species) for session 5. * p < 0.05*

# S15 Results for the reversal learning task

| **Tolerance degree** | **Estimates** | **Std. Error** | **t value** | **Pr (>\|z\|)** |
| --- | --- | --- | --- | --- |
| high - low | -36.11 | 025.21 | -1.433 | 0.331 |
| high - medium | -24.09 | 26.59 | -0.906 | 0.638 |
| low - medium | 12.02 | 24.83 | 0.484 | 0.879 |

*Result of the Tukey HSD test comparing the number of trials to learn the reversed rule for each tolerance degree (low, medium and high tolerance species).*

| **Predictors** | **Estimate** | **Std. Error** | **t value** | **Pr (>\|t\|)** |
| --- | --- | --- | --- | --- |
| **(Intercept)** | 75.979 | 25.187 | 3.017 | 0.004 |
| **age** | 5.092 | 2.105 | 2.419 | 0.019* |
| **sex (male)** | -6.499 | 23.159 | -0.281 | 0.780 |
| **rule (reversed)** | -23.879 | 22.291 | -1.071 | 0.289 |
| **Rank (high ranking)** | 27.857 | 49.785 | 0.560 | 0.581 |

*Results of the LMM for the number of trials to learn the rules (Reversal learning task) in the low tolerance species. Explanatory variables included age, sex, rule (reversed versus acquisition rule), location (Centre of Primatology of the University of Strasbourg vs MRC) and rank (analysis only conducted in females). All full models included the individual ID as a random factor. The estimates (representing the change in the dependent variable relative to the baseline category of each predictor variable), standard error, t-value and p-value are represented.*

| **Predictors** | **Estimate** | **Std. Error** | **t value** | **Pr (>\|t\|)** |
| --- | --- | --- | --- | --- |
| **(Intercept)** | 52.382 | 46.200 | 1.134 | 0.270 |
| **age** | 1.042 | 3.407 | 0.306 | 0.763 |
| **sex (male)** | -21.920 | 20.465 | -1.071 | 0.296 |
| **rule (reversed)** | 34.972 | 16.466 | 2.124 | 0.046* |
| **Rank (high ranking)** | -29.00 | 25.464 | -1.139 | 0.277 |

*Results of the LMM for the number of trials to learn the rules (Reversal learning task) in the species with medium degree of tolerance. Explanatory variables included age, sex, rule (reversed versus acquisition rule) and rank (analysis only conducted in females). All full models included the individual ID as a random factor. The estimates (representing the change in the dependent variable relative to the baseline category of each predictor variable), standard error, t-value and p-value are represented.*

| **Predictors** | **Estimate** | **Std. Error** | **t value** | **Pr (>\|t\|)** |
| --- | --- | --- | --- | --- |
| **(Intercept)** | 50.594 | 43.572 | 35.579 | 1.161 |
| **age** | -0.735 | 2.457 | 35.441 | -0.299 |
| **Sex (male)** | -3.761 | 28.445 | 35.441 | -0.132 |
| **rule (reversed)** | 77.778 | 22.573 | 3.446 | 0.001* |
| **Rank (high ranking)** | -6.745 | 40.404 | -0.167 | 0.869 |

*Results of the GLMM for the accuracy on a No-go trial (Go/No-go task) in high tolerance species. Explanatory included age, sex, rule (reversed versus acquisition rule) and rank (analysis only conducted in males). All full models included the individual ID as a random factor. The estimates (representing the change in the dependent variable relative to the baseline category of each predictor variable), standard error, t-value and p-value are represented.*

*References:*

- Ballesta S, Sadoughi B, Miss F, Whitehouse J, Aguenounon G, Meunier H. (2021) Assessing the reliability of an automated method for measuring dominance hierarchy in non-human primates. Primates. 1-13. https://doi.org/10.1101/2020.11.23.389908
- De Vries H, Stevens JM, Vervaecke H (2006) Measuring and testing the steepness of dominance hierarchies. Anim Behav 71(3):585-592. https://doi.org/10.1016/j.anbehav.2005.05.015
- Elo AE (1987) The rating of chessplayers, past and present. 1^st^ ed., New York: Arco Publication
- Howarth ER, Kemp C, Thatcher HR, Szott ID, Farningham D, Witham CL., ... Bethell EJ (2021) Developing and validating attention bias tools for assessing trait and state affect in animals: A worked example with Macaca mulatta. Appl Anim Behav Sci 234:105198. https://doi.org/10.1016/j.applanim.2020.105198
- Neumann C, Duboscq J, Dubuc C, Ginting A, Irwan AM, Agil M, ... Engelhardt A (2011) Assessing dominance hierarchies: validation and advantages of progressive evaluation with Elo-rating. Anim Behav 82(4):911-921. https://doi.org/10.1016/j.anbehav.2011.07.016
